# The use of corticosteroids for COVID-19 infection

**DOI:** 10.7196/AJTCCM.2020.v26i3.106

**Published:** 2020-07-07

**Authors:** G Richards, C Feldman

**Affiliations:** ^1^ Department of Critical Care, Faculty of Health Sciences, University of the Witwatersrand, Johannesburg, South Africa; ^2^ Department of Internal Medicine, Faculty of Health Sciences, University of the Witwatersrand, Johannesburg, South Africa

**Keywords:** covid-19, coronavirus

## Abstract

The SARS-CoV-2 pandemic is continuing relentlessly in many parts of the world and has resulted in the outpouring of literature on various aspects of the infection, including studies and recommendations regarding the optimal treatment of infected patients. Not surprisingly, the use of corticosteroids in the management of such patients has featured prominently in many of these publications. There is considerable debate in the literature as to the likely benefits, as well as the potential detrimental effects of corticosteroid therapy in general viral respiratory infections and, in particular, COVID-19 infections. While the definitive answer may need to await the results of ongoing randomised, controlled trials recent studies suggest that corticosteroid use in COVID-19 cases with hypoxaemia may benefit from low-dose corticosteroid therapy.

*Afr J Thorac Crit Care Med.* Published online 7 July 2020.

The severe acute respiratory syndrome (SARS)-coronavirus (CoV)-2 pandemic is continuing relentlessly in many parts of the world. In South Africa (SA), for example, as of 29 June 2020, there have been 138 134 cases, as well as 2 456 deaths.[1] Not surprisingly, there has also been an outpouring of literature on various aspects of the infection and multiple official documents have been issued detailing, among other aspects, recommendations for optimal management of this infection in its various stages.[2,3] COVID-19, the disease associated with SARS-CoV-2 infection, manifests in three phases: an upper respiratory tract infection, a pneumonic phase characterised, inter alia, by a rising C-reactive protein (CRP) and hypoxaemia, and a hyperinflammatory phase associated with the acute respiratory distress syndrome (ARDS), multiple organ dysfunction syndrom (MODS), hypercoagulability and shock.[4] Although these phases may merge, and potentially progress from one to another, only a small proportion of patients, overall, appear to progress to the more severe spectrum of disease.

It is difficult to predict precisely who these patients are; however, there are both clinical and laboratory features that indicate a greater likelihood of a worse outcome.[5,6] These include age, obesity, male sex, and comorbidities such as diabetes, hypertension and cardiovascular disease. Laboratory parameters that indicate that progression is occurring are worsening hypoxaemia; rising CRP, D-dimer, ferritin, LDH and interleukin-6 (IL-6) levels; and a declining lymphocyte count. Composites of these have been developed such as the 'immune dysregulation index' in which a ratio of IL-6 to lymphocyte count accurately predicts outcome.[7]

The hyperinflammatory phase correlates with the degree of viraemia with a dramatic increase in IL-6, which is a critical component of the cytokine storm.[8] In other words, patients die not so much from the virus itself but from the immune response to the virus and suppression of this hyperinflammatory response would potentially improve outcome. Therapy should be administered according to disease severity and an anti-inflammatory agent would potentially be of value as an immunomodulatory therapy in those in the pneumonic or hyperinflammatory phases. Ideally this should be accompanied by an effective antiviral agent; however, prior to possibly remdesivir, one has not been available.

The World Health Organization (WHO) document on COVID-19, however, still advocates that systemic corticosteroids (CS) should not be given for treatment of 'viral pneumonia'.[2] They indicate that, given the lack of effectiveness and possible harm, CS should be avoided unless they are indicated for another reason (e.g. exacerbations of asthma or chronic obstructive pulmonary disease (COPD)).[2] An additional document (updated 11 June 2020) summarises the recommendations for all aspects of COVID-19 treatment from various institutions and societies, including the National Institute of Health, the Centers for Disease Control and Prevention (USA), the Infectious Diseases Society of America and others, and provides overall recommendations, including for CS use, according to severity of illness, indications and underlying medical conditions.[3] These institutions indicate that the use of CS in COVID-19 infection is limited and should be carefully considered.

CS have been used for viral infections for some time; however, this remains controversial. We have previously demonstrated benefit with varicella pneumonia;[9] however, more recently studies have shown increased mortality with CS use, for influenza pneumonia.[10-12] Furthermore, although not increasing mortality, a study by Arabi *et al.*[13] showed delayed viral clearance with use of CS in patients with Middle East Respiratory Syndrome (MERS)-CoV. Following this, a recent meta-analysis reviewed outcomes following CS therapy in SARS-CoV-1, MERS-CoV and COVID-19 and also found delayed viral clearance without having a convincing effect on survival, duration of hospitalisation, intensive care units admission, or requirement for mechanical ventilation.[14] This study had limitations; it grouped all potentially lethal coronavirus infections together, and did not qualify at what point the CS were administered, nor whether high or low doses were administered. The authors themselves cautioned that because a preponderance of observational studies was included and because of selection and publication biases, the conclusions, especially regarding SARS-CoV-2, needed to be confirmed by randomised controlled trials (RCT).

It is important to be aware of the fact that people on oral corticosteroids for other indications may be at risk of COVID-19 infection, and increased severity,[15,16] but at the same time, this does not seem to be the case for people on inhaled CS (e.g. for asthma and COPD), for whom recommendations are that they should continue their inhaled CS treatment.[17] In addition, two retrospective studies from China, which noted that CS are often used in patients with COVID-19, reported largely negative results, and potential adverse events from higher doses of systemic CS.[18,19]

Although the WHO has advised against the use of CS in the setting of SARS-CoV-1, influenza and MERS-CoV infection, other studies have, however, demonstrated potential benefit. Two large studies, one of 5 327 patients with SARS-CoV-1[20] and another of 2 141 with H1N1 influenza pneumonia[21] that evaluated the time, dose, and duration of CS reported a significant reduction in mortality with dosage and duration similar to that recommended by the Society of Critical Care Medicine and the European Society of Intensive Care Medicine i.e. low-dose steroids along with low tidal volume ventilation, for non-viral ARDS.[22]

Some studies have also suggested that the use of CS early in the course of COVID-19 may be beneficial, reducing escalation of care and improving outcomes,[23,24] and others suggesting possible benefit in cases with non-COVID-associated ARDS.[25,26] For example, an observational study by Wu *et al.*[25] of patients with CoVID-19 examined risk factors for mortality in patients with ARDS in Wuhan, China, and one of the factors that was associated with significantly improved ($p$=0.003) survival was the administration of methylprednisolone with a hazard ratio (HR) (95% confidence interval (CI)) of 0.38 (0.20 - 0.72).

The Surviving Sepsis Campaign Guideline on COVID-19 suggests low-dose CS therapy in adults with COVID-19 and refractory shock over no CS therapy and suggests against routine CS therapy in adults with COVID-19 and respiratory failure (without ARDS), but suggests CS over not using CS in mechanically ventilated adults with COVID-19 and ARDS.[27]

More recently, a Spanish group conducted a multicentre RCT in 17 ICUs in patients with established, moderate-to-severe non-COVID ARDS (P/F <200 mm Hg) with a positive end-expiratory pressure ≥10 cm $H_2O$ and $FiO_2$ ≥0.5, 24 h after ARDS onset.[28] Patients were randomised to intravenous (IV) dexamethasone 20 mg daily (days 1 - 5), then 10 mg daily (days 6 - 10) or routine care alone. Of 277 patients, 139 were assigned to the dexamethasone group in which ventilator-free days (95% CI) were significantly ($p$<0.0001) higher (4.8 (2.57 - 7.03) days) and 60-day mortality was 21 v. 36% in the controls with a significant ($p$=0.0047) difference of -15.3% (95% CI; -25.9 - -4.9). Adverse events did not differ significantly between groups.

Even more recently, another study demonstrated similar findings.[29] In this single-centre retrospective cohort study of 396 consecutive patients admitted with SARS-CoV-2 pneumonia, those who received CS were compared with patients who did not. The effect of selection bias and potential confounding were adjusted for differences in baseline characteristics by a propensity score, which predicted the probability of being treated with steroids regardless of confounding factors, using multivariable logistic regression. Using this technique, 67 patients were selected as controls. A second propensity score compared different CS regimens. In this study, patients received 1 mg/kg/day methylprednisolone or equivalent, or pulsed CS. Global mortality was 15.1% and the median (IQR) time to CS therapy from symptom onset was 10 (8 - 13) days. The in-hospital mortality was 13.9% in the CS group v. 23.9% in the controls, with an odds ratio (OR) of 0.51 (95% CI; 0.27 - 0.96; $p$=0.044), representing a 41.8% relative risk reduction (RRR) of 0.42 (95% CI; 0.048 - 0.65).

The authors of the most recent study, the as yet unpublished, RECOVERY (Randomised Evaluation of COVID Therapy) RCT reported preliminary findings.[30] Patients with SARS-CoV-2 pneumonia ($n$=2 104) received dexamethasone 6 mg daily orally or intravenously for 10 days and were compared with patients ($n$=4 321) who received usual care alone. The 28-day mortality in the usual care group was 41% in those who required MV, 25% in those requiring oxygen and 13% in those who required neither. In contrast, dexamethasone reduced deaths significantly ($p$=0.0003) in ventilated patients (rate ratio 0.65 (95% CI; 0.48 - 0.88)) and also ($p$=0.0021) in those requiring oxygen only (0.80 (95% CI; 0.67 - 0.96)). Importantly, there was no effect of CS in those who did not require any respiratory support. This translated into the prevention of 1 death per 8 patients on MV and 1 death in 25 patients requiring oxygen alone. While these findings need to be validated by peer review and publication of the study results, they do look very promising.

Given the balance of current evidence, we would suggest the use of low-dose CS in hypoxaemic patients with COVID-19 in an attempt to reduce mortality. Outcomes would potentially be further improved by the addition of an effective antiviral agent, with such a strategy targeting both the viraemia and the secondary systemic inflammatory response.

**Declaration:** None
**Acknowledgements.** None
**Author contributions.** GAR and CF contributed equally.
**Funding.** None
**Conflict of interest.** None

1. SAcoronavirus.co.za (accessed 18 June 2020).
2. World Health Organization. Clinical management of COVID-19 disease: Interim Guidance. Report no WHO/2019-nCoV/clinical/2020.5. Geneva: WHO, 2020. https://www.who.int/publications/i/item/clinical-management-of-covid-19 (accessed 18 June 2020).
3. Assessment of evidence for COVID-19-related treatment: Updated 6/11/2020. https://www.ashp.org/-/media/assets/pharmacy-practice/resource-centers/Coronavirus/docs/ASHP-COVID-19-Evidence-Table.ashx (accessed 18 June 2020).
4. Siddique HK, Mehra MR. COVID-19 illness in native and immunosuppressed states: A clinical-therapeutic staging proposal. J Heart Lung Transplant 2020;39(5)405-407. https://doi.org/10.1016/j.healun.2020.03.012.
5. Intensive Care National Audit and Research Centre. ICNARC report on COVID-19 in critical care 26 June 2020. London: ICNARC, 2020. https://www.icnarc.org/Our-Audit/Audits/Cmp/Reports (accessed 1 July 2020).
6. Huang C, Wang Y, Li X, et al. Clinical features of patients infected with 2019 novel coronavirus in Wuhan, China. Lancet 2020;395(10223):497-506. https://doi.org/10.1016/SO140-6736(20)30183-5.
7. Chen H, Wang J, Su N, et al. Simplified immune-dysregulation index: A novel marker predicts 28-day mortality of intensive care patients with COVID-19. Intensive Care Med 2020;1-3. (epub ahead of print) https://doi.org/10.1007/s00134-020-06114-2.

8. Chen X, Zhao B, Qu Y, et al. Detectable serum SARS-CoV-2 viral load (RNAaemia) is closely correlated with drastically elevated interleukin 6 (IL-6) level in critically ill COVID-19 patients. Clin Infect Dis 2020;ciaa449. (epub ahead of print) https://doi.org/10.1093/cid/ciaa449.
9. Mer M, Richards GA. Corticosteroids in life-threatening varicella pneumonia. Chest 1998;114;426–431. https://doi.org/10.1378/chest.114.2.426.
10. Ni Y-N, Chen G, Sun J, Liang BM, Liang ZA. The effect of corticosteroids on mortality of patients with influenza pneumonia: A systematic review and meta-analysis. Crit Care 2019;23(1):99. https://doi.org/10.1186/s13054-019-2395-8.
11. Moreno G, Rodriguez A, Reyes L-F, et al. Corticosteroid treatment in critically ill patients with severe influenza pneumonia: A propensity score matching study. Intensive Care Med 2018;44(9):1470-1482. https://doi.org/10.1007/s00134-018-5332-4.
12. Delaney JW, Pinto R, Long J, et al. The influence of corticosteroid treatment on the outcome of influenza A (H1N1pdm09)-related critical illness. Crit Care 2016;20:75. https://doi.org/10.1186/s13054-016-1230-8.
13. Arabi YM, Mandourah Y, Al-Hameed F, et al. Corticosteroid therapy for critically ill patients with Middle East Respiratory Syndrome. Am J Respir Crit Care Med 2018;197(6):757-767. https://doi.org/10.1164/rccm.201706-1172OC.
14. Li H, Chen C, Hu F, et al. Impact of corticosteroid therapy on outcomes of persons with SARS-CoV-2, SARS-CoV, or MERS-CoV infection: A systematic review and meta-analysis. Leukemia 2020;34:1503-1511. https//doi.org/10.1038/s41375-020-0848-3.
15. Gianfrancesco M, Hyrich KL, Al-Adely. On behalf of the COVID-19 Global Rheumatology Alliance, et al. Characteristics associated with hospitalisation for COVID-19 in people with rheumatic diseases: Data from the COVID-19 Global Rheumatology Alliance physician-reported registry. Ann Rheum Dis 2020;79:859-866.
16. Brenner EJ, Ungaro RC, Gearry RB, et al. Corticosteroids, but not TNF antagonists, are associated with adverse COVID-19 outcomes in patients with inflammatory bowel diseases: Results from an international registry. Gastroenterology 2020. https://doi.org/10.1053/j.gastro.2020.05.032
17. Halpin MG, Singh D, Hadfield RM. Inhaled corticosteroids and COVID-19: A systematic review and clinical perspective. Eur Respir J 2020;55(5):2001009. https://doi.org/10.1183/13993003.01009-2020.
18. Lu X, Chen T, Wang Y, Wang J, Yan F. Adjuvant corticosteroid therapy for critically ill patients with COVID-19. Crit Care 2020;24:241.
19. Zha L, Li S, Pan L, et al. Corticosteroid treatment of patients with coronavirus disease 2019 (COVID-19). Med J Aust 212(9):416-420. https://doi.org/10.5694/mja2.50577.
20. Long Y, Xu Y, Wang B, et al. Clinical recommendations from an observational study on MERS: Glucocorticoids was benefit in treating SARS patients. Int J Clin Exp Med 2016;9(5):8865–8873.
21. Li H, Yang SG, Gu L, et al. Effect of low-to-moderate-dose corticosteroids on mortality of hospitalized adolescents and adults with influenza A(H1N1)pdm09 viral pneumonia. Influenza Other Respir Viruses 2017;11(4):345-354. https://doi.org/10.1111/irv.12456.
22. Annane D, Pastores SM, Rochwerg B, et al. Guidelines for the diagnosis and management of critical illness-related corticosteroid insufficiency (CIRCI) in critically ill patients (Part I): Society of Critical Care Medicine (SCCM) and European Society of Intensive Care Medicine (ESICM) 2017. Crit Care Med 2017;45(12):2078-2088. https://doi.org/10.1097/CCM.0000000000002737.
23. Fadel R, Morrison AR, Vahia A, et al. Early short course corticosteroids in hospitalized patients with COVID-19. Clin Infect Dis 2020;Ciaa601. https://doi.org/10.1093/cid/ciaa601
24. Russell B, Moss C, Rigg A, Van Hemelrijck M. COVID-19 and treatment with NSAIDs and corticosteroids: Should we be limiting their use in the clinical setting? Ecancermedicalscience 2020;14:1023. htps//doi.org/10.3332/ecancer.2020.1023.
25. Wu C, Chen X, Cai Y, et al. Risk factors associated with acute respiratory distress syndrome and death in patients with coronavirus disease 2019 pneumonia in Wuhan, China. JAMA Intern Med 2020;e200994. https://doi.org/10.1001/jamainternmed.2020.0994.
26. Villar J, Confalonieri M, Pastores SM, Meduri GU. Rationale for prolonged corticosteroid treatment in the acute respiratory distress syndrome caused by coronavirus disease 2019. Crit Care Expl 2020;2(4):e0111. https://doi.org/10.1097/CCE.0000000000000111.
27. Alhazzani W, Moller MH, Arabi YM, et al. Surviving sepsis campaign: Guidelines on the management of critically ill adults with Coronavirus Disease 2019 (COVID-19). Crit Care Med 2020;48(6):e440-e469. https://doi.org/10.1097/CCM.0000000000004363.
28. Villar J, Ferrando C. Dexamethasone treatment for the acute respiratory distress syndrome: A multicentre, randomized controlled trial. Lancet Respir Med 2020;8(3):267-276. https://doi.org/10.1016/S2213-2600(19)30417-5.
29. Cruz AF, Ruiz-Antorán B, Gómez AM, et al. Impact of glucocorticoid treatment in SARS-CoV-2 infection mortality: A retrospective controlled cohort study. Antimicrob Agents Chemother 2020;AAC.01168-20. https://doi.org/10.1128/AAC.01168-20.
30. http://www.ox.ac.uk/news/2020-06-16-dexamethasone-reduces-death-hospitalised-patients-severe-respiratory-complications (accessed 19 June 2020).

*Accepted 29 June 2020.*
